# 4-Carbamoylpyridinium perchlorate

**DOI:** 10.1107/S1600536809039026

**Published:** 2009-10-03

**Authors:** Li-Zhuang Chen

**Affiliations:** a School of Material Science and Engineering, Jiangsu University of Science and Technology, Zhenjiang 212003, People’s Republic of China

## Abstract

In the cation of the title compound, C_6_H_7_N_2_O^+^·ClO_4_^−^, the amide group is oriented at a dihedral angle of 10.41 (17)° to the benzene ring. The crystal structure is stabilized by inter­molecular N—H⋯O hydrogen bonding.

## Related literature

For general background to structural features and physical properties of simple mol­ecular–ionic crystals containing organic cations and acid radicals (1:1 molar ratio), see: Czupiński *et al.* (2002 ▶); Katrusiak & Szafrański (1999 ▶, 2006 ▶). For the crystal structure of 4-carbamoylpyridinium dihydrogen phosphate, see: Gholivand *et al.* (2007 ▶) and for that of 3-(amino­carbon­yl)pyridinium perchlorate, see: Athimoolam & Natarajan (2007 ▶).
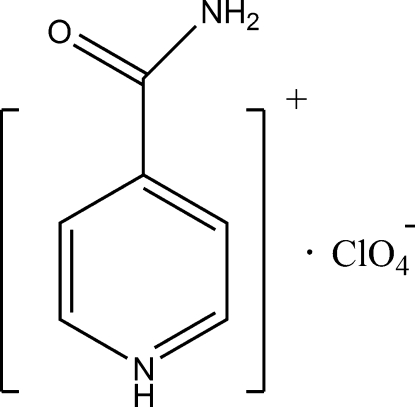


## Experimental

### 

#### Crystal data

C_6_H_7_N_2_O^+^·ClO_4_^−^
*M* *_r_* = 222.59
Monoclinic, 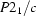

*a* = 10.935 (2) Å
*b* = 10.082 (2) Å
*c* = 8.2021 (16) Å
β = 99.37 (3)°
*V* = 892.2 (3) Å^3^
*Z* = 4
Mo *K*α radiation
μ = 0.43 mm^−1^
*T* = 293 K
0.30 × 0.25 × 0.22 mm

#### Data collection

Rigaku SCXmini diffractometer
Absorption correction: multi-scan (*CrystalClear*; Rigaku, 2005 ▶) *T*_min_ = 0.87, *T*_max_ = 0.90
8863 measured reflections
2033 independent reflections
1421 reflections with *I* > 2σ(*I*)
*R*_int_ = 0.074

#### Refinement

*R*[*F* ^2^ > 2σ(*F* ^2^)] = 0.064
*wR*(*F* ^2^) = 0.176
*S* = 1.04
2033 reflections
127 parameters
H-atom parameters constrained
Δρ_max_ = 0.42 e Å^−3^
Δρ_min_ = −0.42 e Å^−3^

### 

Data collection: *CrystalClear* (Rigaku, 2005 ▶); cell refinement: *CrystalClear*; data reduction: *CrystalClear*; program(s) used to solve structure: *SHELXS97* (Sheldrick, 2008 ▶); program(s) used to refine structure: *SHELXL97* (Sheldrick, 2008 ▶); molecular graphics: *ORTEP-3 for Windows* (Farrugia, 1997 ▶); software used to prepare material for publication: *WinGX* (Farrugia, 1999 ▶).

## Supplementary Material

Crystal structure: contains datablocks I, global. DOI: 10.1107/S1600536809039026/xu2591sup1.cif

Structure factors: contains datablocks I. DOI: 10.1107/S1600536809039026/xu2591Isup2.hkl

Additional supplementary materials:  crystallographic information; 3D view; checkCIF report

## Figures and Tables

**Table 1:** Hydrogen-bond geometry (Å, °)

*D*—H⋯*A*	*D*—H	H⋯*A*	*D*⋯*A*	*D*—H⋯*A*
N1—H1*A*⋯O3^i^	0.86	2.10	2.932 (4)	162
N2—H2*A*⋯O1^ii^	0.86	2.32	3.162 (4)	168
N2—H2*B*⋯O5^iii^	0.86	2.17	3.004 (4)	164

Symmetry codes: (i) 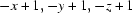; (ii) 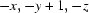; (iii) 
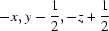
.

**Fig. 1.**
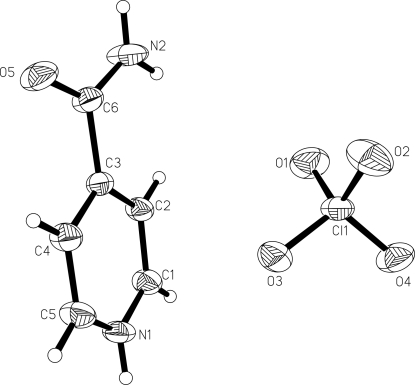
The asymmetric unit of the title compound with atom labels. Displacement ellipsoids were drawn at the 30% probability level.

## supplementary crystallographic information

### Comment

Recently much attention has been devoted to simple molecular–ionic crystals containing organic cations and acid radicals (1:1 molar ratio) due to the tunability of their special structural features and their interesting physical properties (Czupiński *et al.*, 2002; Katrusiak & Szafrański, 1999; Katrusiak & Szafrański, 2006). The crystal structures of 4-carbamoylpyridinium dihydrogen phosphate (Gholivand *et al.*, 2007) and 3-(aminocarbonyl)pyridinium perchlorate (Athimoolam & Natarajan, 2007) have been reported previously. In our laboratory, a compound containing 4-carbamoylpyridinium cation and ClO_4_^-^ anion has been synthesized, its crystal structure is reported herein.

The asymmetric unit of the title compound (Fig. 1) consists of one 4-carbamoylpyridinium cation and one ClO_4_^-^ anion. The crystal structure is stabilized by intermolecular N—H···O hydrogen bonds (Table 1).

### Experimental

4-Carbamoylpyridine (2.44 g, 20 mmol) and 10% aqueous solution (15 ml) of HClO_4_ were dissolved in 30 ml water. The solution was heated at 343 K for 0.5 h, forming a clear solution. The reaction mixture was cooled slowly to room temperature, block crystals of the title compound were formed.

### Refinement

All H atoms were placed in calculated positions with C—H = 0.93 and N—H = 0.86 Å, and refined using a riding model with U_iso_(H) = 1.2U_eq_(C,N).

### Crystal data

**Table:**

C_6_H_7_N_2_O^+^·ClO_4_^−^	*F*(000) = 456
*M**_r_* = 222.59	*D*_x_ = 1.657 Mg m^−^^3^
Monoclinic, *P*2_1_/*c*	Mo *K*α radiation, λ = 0.71073 Å
Hall symbol: -P 2ybc	Cell parameters from 1421 reflections
*a* = 10.935 (2) Å	θ = 3.2–27.5°
*b* = 10.082 (2) Å	µ = 0.43 mm^−^^1^
*c* = 8.2021 (16) Å	*T* = 293 K
β = 99.37 (3)°	Block, colorless
*V* = 892.2 (3) Å^3^	0.30 × 0.25 × 0.22 mm
*Z* = 4	

### Data collection

**Table:**

Rigaku SCXmini diffractometer	2033 independent reflections
Radiation source: fine-focus sealed tube	1421 reflections with *I* > 2σ(*I*)
graphite	*R*_int_ = 0.074
Detector resolution: 13.6612 pixels mm^-1^	θ_max_ = 27.5°, θ_min_ = 3.2°
ω scans	*h* = −14→14
Absorption correction: multi-scan (*CrystalClear*; Rigaku, 2005)	*k* = −13→13
*T*_min_ = 0.87, *T*_max_ = 0.90	*l* = −10→10
8863 measured reflections	

### Refinement

**Table:**

Refinement on *F*^2^	Primary atom site location: structure-invariant direct methods
Least-squares matrix: full	Secondary atom site location: difference Fourier map
*R*[*F*^2^ > 2σ(*F*^2^)] = 0.064	Hydrogen site location: inferred from neighbouring sites
*wR*(*F*^2^) = 0.176	H-atom parameters constrained
*S* = 1.04	*w* = 1/[σ^2^(*F*_o_^2^) + (0.0844*P*)^2^ + 0.3072*P*] where *P* = (*F*_o_^2^ + 2*F*_c_^2^)/3
2033 reflections	(Δ/σ)_max_ < 0.001
127 parameters	Δρ_max_ = 0.42 e Å^−^^3^
0 restraints	Δρ_min_ = −0.42 e Å^−^^3^

### Special details

**Table:**

Geometry. All e.s.d.'s (except the e.s.d. in the dihedral angle between two l.s. planes) are estimated using the full covariance matrix. The cell e.s.d.'s are taken into account individually in the estimation of e.s.d.'s in distances, angles and torsion angles; correlations between e.s.d.'s in cell parameters are only used when they are defined by crystal symmetry. An approximate (isotropic) treatment of cell e.s.d.'s is used for estimating e.s.d.'s involving l.s. planes.
Refinement. Refinement of *F*^2^ against ALL reflections. The weighted *R*-factor *wR* and goodness of fit *S* are based on *F*^2^, conventional *R*-factors *R* are based on *F*, with *F* set to zero for negative *F*^2^. The threshold expression of *F*^2^ > σ(*F*^2^) is used only for calculating *R*-factors(gt) *etc*. and is not relevant to the choice of reflections for refinement. *R*-factors based on *F*^2^ are statistically about twice as large as those based on *F*, and *R*- factors based on ALL data will be even larger.

### Fractional atomic coordinates and isotropic or equivalent isotropic displacement parameters (Å^2^)

**Table:**

	*x*	*y*	*z*	*U*_iso_*/*U*_eq_	
Cl1	0.35049 (7)	0.39747 (7)	0.09900 (9)	0.0473 (3)	
O5	0.0368 (2)	0.8048 (2)	0.2309 (3)	0.0650 (7)	
N2	−0.0251 (3)	0.5945 (3)	0.1873 (4)	0.0698 (10)	
H2A	−0.0862	0.6169	0.1127	0.084*	
H2B	−0.0128	0.5123	0.2130	0.084*	
C6	0.0493 (3)	0.6856 (3)	0.2619 (4)	0.0463 (7)	
C4	0.2469 (3)	0.7332 (3)	0.4470 (4)	0.0479 (8)	
H4A	0.2386	0.8205	0.4104	0.057*	
N1	0.3573 (2)	0.5717 (3)	0.6122 (3)	0.0541 (8)	
H1A	0.4218	0.5492	0.6813	0.065*	
C3	0.1569 (2)	0.6406 (3)	0.3883 (3)	0.0399 (7)	
O4	0.4364 (2)	0.2906 (2)	0.1204 (3)	0.0707 (8)	
O3	0.3944 (2)	0.4992 (2)	0.2176 (3)	0.0653 (7)	
C2	0.1692 (3)	0.5117 (3)	0.4485 (4)	0.0457 (8)	
H2C	0.1096	0.4479	0.4117	0.055*	
C5	0.3479 (3)	0.6961 (4)	0.5590 (4)	0.0549 (9)	
H5A	0.4094	0.7575	0.5973	0.066*	
O2	0.3407 (3)	0.4505 (3)	−0.0636 (3)	0.0843 (9)	
O1	0.2315 (2)	0.3517 (3)	0.1231 (4)	0.0759 (8)	
C1	0.2713 (3)	0.4798 (3)	0.5636 (4)	0.0536 (9)	
H1B	0.2803	0.3945	0.6072	0.064*	

### Atomic displacement parameters (Å^2^)

**Table:**

	*U*^11^	*U*^22^	*U*^33^	*U*^12^	*U*^13^	*U*^23^
Cl1	0.0405 (5)	0.0430 (5)	0.0519 (5)	0.0012 (3)	−0.0116 (3)	0.0000 (3)
O5	0.0581 (15)	0.0403 (14)	0.0867 (19)	0.0094 (11)	−0.0176 (12)	0.0095 (12)
N2	0.0575 (19)	0.0488 (18)	0.087 (2)	−0.0020 (13)	−0.0377 (17)	0.0116 (15)
C6	0.0397 (17)	0.0405 (18)	0.0548 (19)	0.0032 (13)	−0.0039 (13)	0.0050 (14)
C4	0.0466 (18)	0.0361 (16)	0.057 (2)	−0.0056 (13)	−0.0036 (14)	0.0033 (13)
N1	0.0460 (16)	0.0565 (18)	0.0509 (17)	0.0061 (12)	−0.0187 (13)	−0.0010 (12)
C3	0.0346 (16)	0.0371 (15)	0.0448 (17)	0.0021 (11)	−0.0030 (12)	−0.0012 (12)
O4	0.0550 (15)	0.0510 (15)	0.097 (2)	0.0147 (11)	−0.0145 (14)	−0.0040 (13)
O3	0.0595 (16)	0.0570 (15)	0.0721 (17)	−0.0015 (11)	−0.0110 (12)	−0.0208 (12)
C2	0.0469 (18)	0.0319 (15)	0.0520 (19)	−0.0018 (12)	−0.0109 (14)	−0.0034 (12)
C5	0.0438 (19)	0.055 (2)	0.060 (2)	−0.0120 (15)	−0.0079 (15)	−0.0007 (16)
O2	0.106 (2)	0.087 (2)	0.0518 (17)	−0.0028 (17)	−0.0107 (15)	0.0126 (14)
O1	0.0434 (15)	0.0839 (19)	0.096 (2)	−0.0120 (13)	−0.0021 (13)	−0.0088 (16)
C1	0.060 (2)	0.0381 (17)	0.055 (2)	0.0063 (15)	−0.0145 (16)	−0.0002 (14)

### Geometric parameters (Å, °)

**Table:**

Cl1—O4	1.421 (2)	C4—C3	1.385 (4)
Cl1—O2	1.424 (3)	C4—H4A	0.9300
Cl1—O1	1.425 (3)	N1—C5	1.327 (4)
Cl1—O3	1.441 (2)	N1—C1	1.334 (4)
O5—C6	1.231 (3)	N1—H1A	0.8600
N2—C6	1.310 (4)	C3—C2	1.389 (4)
N2—H2A	0.8600	C2—C1	1.377 (4)
N2—H2B	0.8600	C2—H2C	0.9300
C6—C3	1.507 (4)	C5—H5A	0.9300
C4—C5	1.369 (5)	C1—H1B	0.9300
			
O4—Cl1—O2	110.33 (19)	C5—N1—C1	123.0 (3)
O4—Cl1—O1	109.76 (17)	C5—N1—H1A	118.5
O2—Cl1—O1	108.68 (18)	C1—N1—H1A	118.5
O4—Cl1—O3	108.36 (15)	C4—C3—C2	119.0 (3)
O2—Cl1—O3	109.30 (18)	C4—C3—C6	117.9 (3)
O1—Cl1—O3	110.41 (17)	C2—C3—C6	123.1 (3)
C6—N2—H2A	120.0	C1—C2—C3	118.9 (3)
C6—N2—H2B	120.0	C1—C2—H2C	120.6
H2A—N2—H2B	120.0	C3—C2—H2C	120.6
O5—C6—N2	123.2 (3)	N1—C5—C4	119.3 (3)
O5—C6—C3	118.9 (3)	N1—C5—H5A	120.3
N2—C6—C3	117.8 (3)	C4—C5—H5A	120.3
C5—C4—C3	120.0 (3)	N1—C1—C2	119.7 (3)
C5—C4—H4A	120.0	N1—C1—H1B	120.1
C3—C4—H4A	120.0	C2—C1—H1B	120.1
			
C5—C4—C3—C2	1.9 (5)	C4—C3—C2—C1	−0.6 (5)
C5—C4—C3—C6	−178.4 (3)	C6—C3—C2—C1	179.7 (3)
O5—C6—C3—C4	−9.0 (5)	C1—N1—C5—C4	−1.0 (5)
N2—C6—C3—C4	169.5 (3)	C3—C4—C5—N1	−1.2 (5)
O5—C6—C3—C2	170.7 (3)	C5—N1—C1—C2	2.4 (5)
N2—C6—C3—C2	−10.9 (5)	C3—C2—C1—N1	−1.5 (5)

### Hydrogen-bond geometry (Å, °)

**Table:**

*D*—H···*A*	*D*—H	H···*A*	*D*···*A*	*D*—H···*A*
N1—H1A···O3^i^	0.86	2.10	2.932 (4)	162
N2—H2A···O1^ii^	0.86	2.32	3.162 (4)	168
N2—H2B···O5^iii^	0.86	2.17	3.004 (4)	164

Symmetry codes: (i) −*x*+1, −*y*+1, −*z*+1; (ii) −*x*, −*y*+1, −*z*; (iii) −*x*, *y*−1/2, −*z*+1/2.
